# Test–retest reliability of eye tracking measures in a computerized Trail Making Test

**DOI:** 10.1167/jov.23.8.15

**Published:** 2023-08-18

**Authors:** Lukas Recker, Christian H. Poth

**Affiliations:** 1 Neuro-Cognitive Psychology and Center for Cognitive Interaction Technology, Bielefeld University, Bielefeld, Germany; 2 Neuro-Cognitive Psychology and Center for Cognitive Interaction Technology, Bielefeld University, Bielefeld, Germany

**Keywords:** intraclass correlation, Bland–Altman, neuropsychological assessment, individual differences

## Abstract

The Trail Making Test (TMT) is a frequently applied neuropsychological test that evaluates participants’ executive functions based on their time to connect a sequence of numbers (TMT-A) or alternating numbers and letters (TMT-B). Test performance is associated with various cognitive functions ranging from visuomotor speed to working memory capabilities. However, although the test can screen for impaired executive functioning in a variety of neuropsychiatric disorders, it provides only little information about which specific cognitive impairments underlie performance detriments. To resolve this lack of specificity, recent cognitive research combined the TMT with eye tracking so that eye movements could help uncover reasons for performance impairments. However, using eye-tracking-based test scores to examine differences between persons, and ultimately apply the scores for diagnostics, presupposes that the reliability of the scores is established. Therefore, we investigated the test–retest reliabilities of scores in an eye-tracking version of the TMT recently introduced by Recker et al. (2022). We examined two healthy samples performing an initial test and then a retest 3 days (*n* = 31) or 10 to 30 days (*n* = 34) later. Results reveal that, although reliabilities of classic completion times were overall good, comparable with earlier versions, reliabilities of eye-tracking-based scores ranged from excellent (e.g., durations of fixations) to poor (e.g., number of fixations guiding manual responses). These findings indicate that some eye-tracking measures offer a strong basis for assessing interindividual differences beyond classic behavioral measures when examining processes related to information accumulation processes but are less suitable to diagnose differences in eye–hand coordination.

## Introduction

To assess neurocognitive functionality, neuropsychologists challenge individuals in certain aspects of their performance using neuropsychological tests. Using these tests for neuropsychological diagnostics in applied research and clinical settings requires that measures of test performance can be obtained and interpreted efficiently and easily. Therefore, most readily available measures, such as reaction times and errors, are the variables most frequently used to derive test scores quantifying performance. One test widely used as an easily applicable screening of cognitive performance is the Trail Making Test (TMT) (Reitan, 1958). In this test, participants connect a sequence of numbers (TMT-A) or alternating numbers and letters (TMT-B) via pencil on paper. Based on the time needed to complete the sequence, the participant’s level of executive functioning is evaluated (Bowie & Harvey, 2006; Salthouse, 2011; Sánchez-Cubillo et al., 2009). This powerful but broad measure is frequently applied to examine and diagnose multiple neuropsychiatric disorders (e.g., Ashendorf, Jefferson, O'Connor, Chaisson, Green, & Stern, 2008; Muir et al., 2015; O'Rourke et al., 2011; Wölwer & Gaebel, 2002) or investigate executive functions of healthy individuals (e.g., Hwang et al., 2016). However, a number of different cognitive functions are implicated in completing the sequence, so that completion time cannot differentiate between specific neurocognitive processes that underlie performance. To address this problem, recent studies have introduced measures beyond completion times that more specifically reflect cognitive processes and mechanisms and therefore offer a more detailed understanding of impaired and intact cognitive functionality. That is, they introduced a computerized version of the test that included eye tracking (Linari, Juantorena, Ibáñez, Petroni, & Kamienkowski, 2022; Recker, Foerster, Schneider, & Poth, 2022; Wölwer & Gaebel, 2002). This addition allows, for example, assessing the number and length of participants’ eye fixations or the amplitude of their saccadic eye movements, both of which offer new information about cognitive processes, such as attentional selection of visual information and the allocation of cognitive processing resources (e.g., Hutton, 2008; Liversedge & Findlay, 2000; Salthouse & Ellis, 1980). Ongoing technological advancements such as portable eye trackers or virtual reality goggles (Foerster, Poth, Behler, Botsch, & Schneider, 2016; Foerster, Poth, Behler, Botsch, & Schneider, 2019) and their combination with eye tracking make it more and more feasible to apply experimental tests with eye tracking in neuropsychological testing scenarios. In the context of the TMT, several recent studies have already demonstrated the utility of eye tracking to understand task performance in terms of specific cognitive functions (Linari et al., 2022; Recker et al., 2022; Wölwer & Gaebel, 2002). However, to use such new measures for individual diagnostics in research and applied settings, it is important to first establish that the measures are reliable (Mollon, Bosten, Peterzell, & Webster, 2017; Wilmer, 2017). This seems particularly important, because the reliability determines how strong the measures could maximally correlate with measures from other tests or diagnostic assessments (e.g., neurological assessments). As such, the reliability of the measures lays a necessary foundation for later validations based on correlations with other tests and clinical diagnostics (Cronbach & Meehl, 1955). Therefore, here we aim to assess the test–retest reliability of a computerized TMT including a detailed neurocognitive profile of different eye-tracking-based measures first introduced by Recker et al. (2022).

The traditionally conducted TMT is a paper-and-pencil test that assesses the level of executive functioning of participants. It is made up of two parts, each thought to reflect a set of different cognitive functions. The TMT-A consists of a sequence of numbers from 1 to 25 which participants are asked to connect in ascending order. Part A of the test is associated with measures of processing and motor speed and visual search (Bowie & Harvey, 2006; Crowe, 1998; Salthouse, 2011; Sánchez-Cubillo et al., 2009). The TMT-B consists of a sequence of numbers from 1 to 13 and letters from A to L which participants are asked to connect in ascending order alternating between the two sets. Part B is associated with measures of task switching and cognitive flexibility (Arbuthnott & Frank, 2000; Bowie & Harvey, 2006; Kortte, Horner, & Windham, 2002; Sánchez-Cubillo et al., 2009). Deviations in completion times and errors in both test halves can therefore be a useful indicator of impairments in the executive functions of patients, also supported by matching neural correlates revealed by a number of lesion studies (Kopp et al., 2015; Muir et al., 2015; Reitan, 1958; Varjacic, Mantini, Demeyere, & Gillebert, 2018). Executive functions cover a range of cognitive domains influencing our everyday behavior (Cohen, 2017; Diamond, 2013). However, in contrast to the range of affected functions, the TMT is limited by its restricted set of outcome measures; therefore, conclusions about impairments of cognitive functions are rather unspecific. For example, slowed performance in a sequence can stem from multiple sources of subprocesses, such as difficulties with locating targets, tracking the current position, performing the actual movement, etc. Amending the test with the capabilities provided by eye tracking can shed light on subprocesses influencing the overall performance. Eye movements are closely linked to perceptual and attentional processes (Findlay & Gilchrist, 2008; Land & Tatler, 2009; Schütz, Braun, & Gegenfurtner, 2011) and are grounded by a variety of cognitive (Bundesen, Habekost, & Kyllingsbæk, 2011; Hutton, 2008; Wolfe, 2021) and neurophysiological (Henderson & Choi, 2015; Krauzlis, Goffart, & Hafed, 2017; Schall & Thompson, 1999) theories. Therefore, investigating eye movements can help discriminate reasons for emerging patterns in performances and thus increase the conceptual specificity of a test.

Up until now, only a few studies have been conducted applying eye tracking in the context of the TMT. Next to a study using eye movements as a substitute for hand movements in patient groups with motor impairments (Hicks et al., 2013), so far to the best of our knowledge only three studies have analyzed eye movements in computerized versions of the TMT while still using manual actions to perform the task. To disentangle the impaired performance of schizophrenic patients in the TMT (e.g., Laere, Tee, & Tang, 2018; Periáñez et al., 2007), Wölwer and Gaebel (2002) conducted a study using a computerized TMT while also recording participants’ gaze. Analyzing the spatial alignment of cursor and eye positions they found that differences in performance (i.e., completion times) were the result of differences in what they called “planning periods,” periods spent planning the next movement as opposed to periods monitoring the current mouse cursor movement. That is, schizophrenic patients differed in the time and efficiency spent planning their movements to the next target. Linari et al. (2022) further expanded the examined eye movement measures including saccade durations and saccade amplitude. However, they found that, of the examined eye movement measures, only the number of fixations differed between TMT-A and TMT-B. Segregating task performance into periods, similar to Wölwer and Gaebel (2002), they found that the difference in the number of fixations came from a prolonged period of exploration and planning in the TMT-B. Finally, in a recent study, Recker et al. (2022) introduced a computerized TMT that adds eye-tracking measures more commonly applied in natural task studies, such as different fixation types and the eye–hand span (e.g., Foerster, Carbone, Koesling, & Schneider, 2011; Land & Hayhoe, 2001; Land, Mennie, & Rusted, 1999). They also found differences in the number of fixations between test halves A and B. Analyses of the fixation types revealed that these differences came from a change in searching fixations (i.e., fixations on previous or future targets) as opposed to guiding fixations (i.e., fixations on the current target). Taken together, these studies highlight the additional insight into more specific cognitive processes provided by eye tracking in the TMT.

In their study, Recker et al. (2022) also included an additional manipulation of participants’ task set. They instructed participants to perform both test halves once emphasizing speed and once emphasizing accuracy. Using this manipulation, they investigated which of their included test scores was relatively (in-)dependent of this factor, granting additional specificity in terms of their relationship with cognitive control. In the original TMT, the difference in completion times between test halves A and B is frequently calculated to provide a score associated with the cognitive control abilities of participants (Sánchez-Cubillo et al., 2009). Using the newly included manipulation of emphasizing speed or accuracy introduces the possibility of providing an additional measure of cognitive control. In contrast to the difference score, which depends on a stimulus-driven contrast between conditions (i.e., sequence of numbers vs. sequence of alternating numbers and letters), this measure is based solely on an internal switch between task sets in TMT-A and TMT-B. The ability to shift between the emphasis on speed or accuracy seems to present one of the most fundamental internal priorities we can set affecting almost any given task (Carrasco & McElree, 2001; Dietze & Poth, 2022; Heitz, 2014; Rae, Heathcote, Donkin, Averell, & Brown, 2014; Wickelgren, 1977). Calculating a score based on this internal criterion can therefore extend the capabilities of the TMT to measure abilities of cognitive control without the need for further stimulus-dependent alternations of the task.

To not only improve the general understanding of performance-determining mechanism but also use newly introduced scores for the study of interindividual differences, it is necessary to assess such scores in terms of their test–retest reliability (Goodhew & Edwards, 2019; Mollon et al., 2017; Wilmer, 2008). In the past, most common approaches in vision science and experimental psychology have put an emphasis on describing effects for the “standard observer.” That is, variance introduced by the individual would be discarded as noise and dismissed for further analyses to focus on the effects of experimental conditions and their related cognitive processes. Paradigms that have emerged from this research tradition often are designed to maximize experimental effects, and little is known about the reliability of their respective eye-tracking measures. Recent studies have shown that some paradigms produce stable, replicable results but are not suited for the examination of interindividual differences (Clark, Birch-Hurst, Pennington, Petrie, Lee, & Hedge, 2022; Hedge, Powell, & Sumner, 2018). On the other hand, other research investigating eye-tracking measures for the study of interindividual differences has provided promising results. An early study by Andrews and Coppola (1999) found that fixation durations and saccadic amplitudes correlated in two clusters of tasks, which they framed as active and passive. This stability across tasks was repeatedly demonstrated (e.g., Boot, Becic, & Kramer, 2009; Castelhano, Mack, & Henderson, 2009; Rayner, Li, Williams, Cave, & Well, 2007) and later found to also withstand time (Henderson & Luke, 2014). In the reading literature, there has been an effort to demonstrate the reliability and stability of different eye movement measures over time (Carter & Luke, 2018; Henderson & Luke, 2014; Staub, 2021). An extensive study by Bargary, Bosten, Goodbourn, Lawrance-Owen, Hogg, and Mollon (2017) suggested that eye-tracking measures can provide an idiosyncratic signature by which individuals could be identified because of high interindividual stability. These studies illustrate on the one hand the potential to use eye tracking for the study of interindividual differences while on the other hand stressing the importance of first establishing their reliabilities.

Because the reliabilities of eye tracking and related additional scores in the TMT are largely unknown, we want to focus on this research question. In the present study, we therefore examine the test–retest reliability of the test scores included and introduced by Recker et al. (2022) in their computer-based version of the TMT. Furthermore, we include a new additional test score describing the speed–accuracy tradeoff of participants as an internal measure of cognitive control in the examination of executive functions. We examine two samples each collected across two sessions spanning multiple days. Sample A contains a retest after 3 days of initial testing. Sample B contains a retest after 10 to 30 days of initial testing. The different lag times allow an inspection of relatively short- and mid-term reliability and stability of our scores. For example, training effects could affect performances with repeated testing. Different lag times allow us to see whether and how long these effects might persist, and if they differ between test intervals how this affects reliability. To analyze, we calculated the intraclass correlation coefficient (ICC) as an indicator of test–retest reliability and utilized additional measures of agreement based on Bland–Altman calculations (Bland & Altman, 1999; Haghayegh, Kang, Khoshnevis, Smolensky, & Diller, 2020). The ICC indicates whether we can consider a measure to be relatively stable. A high ICC indicates that differences between persons are stable across time points; that is, their respective position (rank) is stable within a reference group but gives no information about whether absolute values changed between measurements. Therefore, we included metrics of Bland–Altman calculations that indicate whether and how much the absolute values changed. This becomes important when single individuals are tested repeatedly, such as to investigate improvements from one point to the next which is often relevant in clinical settings. We expect results on completion times in the new version of the TMT to resemble the reliabilities of the original version of the test. Previous work on eye movements in the TMT (Hicks et al., 2013; Linari et al., 2022; Recker et al., 2022) and a variety of different cognitive tasks (e.g., Andrews & Coppola, 1999; Henderson & Luke, 2014; Rayner et al., 2007) found that fixation durations and saccade amplitudes were relatively stable across examined conditions and tasks. This might also be reflected in high stabilities across sessions. As for the rest of the eye tracking-related measures, it is unknown whether previous differences between conditions also produce stable individual differences between persons.

## Methods

### Participants

The dataset is divided into two samples gathered across two larger research projects. In both projects, the experiment was conducted as part of a selection of multiple experiments. Within the first project (sample A), 31 healthy participants (26 female, five male) were tested in two sessions spread 3 days apart. Participants in this group were between 19 and 38 years old (median = 23; interquartile range [IQR] = 4) and tested for normal or corrected-to-normal vision. Within the second project (sample B), 34 healthy participants (24 female, 10 male) were tested. Due to the Covid-19 pandemic, participants completed their second session with variable intervals between sessions (i.e., 10–30 days).^1^ On average, the time between sessions was 16 days. Participants in this group were between 18 and 53 years old (median = 24; IQR = 4) and were also tested for normal or corrected-to-normal vision.

We report and calculated ICCs for sample A and sample B individually. That is, calculations are based on measurements of sessions one and two of the respective sample.

The analysis of the dataset as part of this study was preregistered via the Open Science Framework (https://osf.io/fwhjb/). Participants gave written informed consent before their participation and received course credit or monetary compensation as a reward. The study was approved by the local ethics committee of Bielefeld University within the context of both larger research projects.

### Apparatus and stimuli

The apparatus, stimuli, and procedures were the same (same lab facilities, same experimental code) for the samples in both projects. Participants sat down in a dimly lit room in front of a ViewSonic G90FB CRT monitor (ViewSonic, Brea, CA) with a resolution of 1024 × 768 pixels (corresponding to 36 × 27 cm physical dimensions) with a refresh rate of 100 Hz (preheated for at least 5 minutes (Poth & Horstmann, 2017). Each participant's head position was stabilized using a chin-rest placed 71 cm away from the screen. They controlled the experiment handling a Logitech RX250 computer mouse (Logitech, Lausanne, Switzerland) with their right hand. The position of the mouse was sampled with 100 Hz. Movements of their right eye were recorded with a sample rate of 1000 Hz via a tower-mounted EyeLink 1000 Eye Tracker (SR Research, Ottawa, ON, Canada). The experiment ran on a Windows 7 desktop PC (Microsoft, Redmond, WA) and was written in Python 3.6 using packages psychopy (Peirce et al., 2019) and pylink (SR Research) for stimulus presentation and control of the eye tracker, respectively.

Stimuli appeared on a uniform, gray background (30 cd/cm^2^). Targets were black, unfilled circles with 1.35° diameter. Numbers or letters making up the sequence were presented within the circles and written in black, using the Arial font set to 20 points for the number/letter height. When a target was hit correctly (i.e., the participant clicked the current target within the circle), it changed its color from black (1.5 cd/cm^2^) to white (93.2 cd/cm^2^). In each trial, stimuli were randomly placed across the screen using a 5 × 5 grid with cells being 4.32° × 4.32° in size. Each grid cell contained one stimulus, and the minimum distance between stimuli was defined to be at least 1.35°.

### Procedure

**Figure 1.**
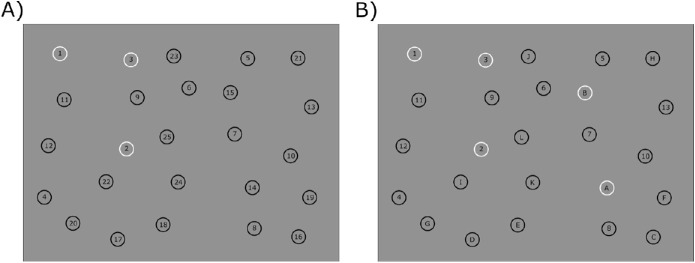
Example stimulus displays for TMT-A (A) and TMT-B (B).

Participants received written on-screen instructions on the overall task of clicking through a sequence of numbers (TMT-A) or numbers and letters (TMT-B). Per session, each participant completed 10 trials divided into two blocks. They first completed five trials of TMT-A and then five trials of TMT-B, including one shortened training trial (eight targets) each. Before each block they completed a nine-point grid calibration for the eye tracker. Before each trial, they were instructed on-screen to complete the next sequence as quickly or as accurately as possible (i.e., hit the targets as centrally as possible). The order of blocks was the same for all participants (e.g., first TMT-A, then TMT-B), and the order of instructions (e.g., speed or accuracy emphasis) was randomized across participants. Each instruction was given two times per block; that is, participants completed two trials emphasizing speed and two trials emphasizing accuracy in TMT-A and TMT-B, respectively. They then started the trial by simultaneously looking at and clicking on a centrally presented fixations cross. Subsequently, they clicked through the presented sequence of 25 numbers (1, 2, 3, …, 25) or 25 alternating numbers and letters (1, A, 2, B, …, 13) (see Figure 1 for examples of stimulus displays). If they hit a target, its color changed from black to white. If they missed a target, the circumcircle remained black, indicating that the number was not successfully checked. Clicks were counted as correct if they fell within the circumcircle of the number/ letter. Note that no paths were drawn on the screen, contrary to original versions of the TMT. This also allowed for paths between targets to cross. Participants only clicked through the sequence. Overall, the experiment took approximately 10 minutes. During their second sessions participants performed the exact same experiment again; that is, the order of trials (e.g., instructions) and spatial distributions of the targets were the same as during session one.

### Dependent variables

We included the following dependent variables in our analyses of reliabilities: The *completion time* of participants was defined as the time between initiation of a trial (i.e., clicking the fixation stimulus) and the first click on the last target of a sequence. The *speed–accuracy measure* represents the slope of regressing the reaction time per target on the accuracy of the click relative to the center of that target. For this purpose, we determined the relative accuracy of a click between 1 (i.e., target hit exactly in the center of its circumcircle) and 0 (i.e., target missed). Predicting this accuracy with the reaction times of the respective targets allowed us to compare speed–accuracy trade-offs between experimental conditions via their slopes (Heitz, 2014). Based on the EyeLink algorithm, fixations and saccades are detected using a velocity threshold of 30° × s^−1^ and an acceleration threshold of 8000° × s^−2^. Blinks and events preceding or following a blink by 50 ms were excluded. We used median *fixation durations* and the overall *number of fixations* for our analysis, excluding fixations shorter than 50 ms. The number of fixations was further broken down into the number of searching and the number of guiding fixations. The *number of searching fixations* is given by all fixations that fall on an object that is not the current target of the sequence. In contrast, the *number of guiding fixations* describes the number of fixations on current targets of the sequence (Foerster & Schneider, 2015; Land & Tatler, 2009). Either way, fixations were counted as falling on an object if they were within a 3.25° radius of an object (the target). In terms of saccades, we used the median *saccade amplitude*, excluding saccades shorter than 0.1° to avoid microsaccades (Martinez-Conde, Macknik, Troncoso, & Hubel, 2009). Finally, we looked at the *scanpath length* as the overall path the eyes covered during a trial and calculated the *eye–hand span* as the time between a fixation on a target and the proceeding click (Land & Tatler, 2009). Positive values in this measure indicate that the eyes led the hand.

### Statistical analysis

**Figure 2.**
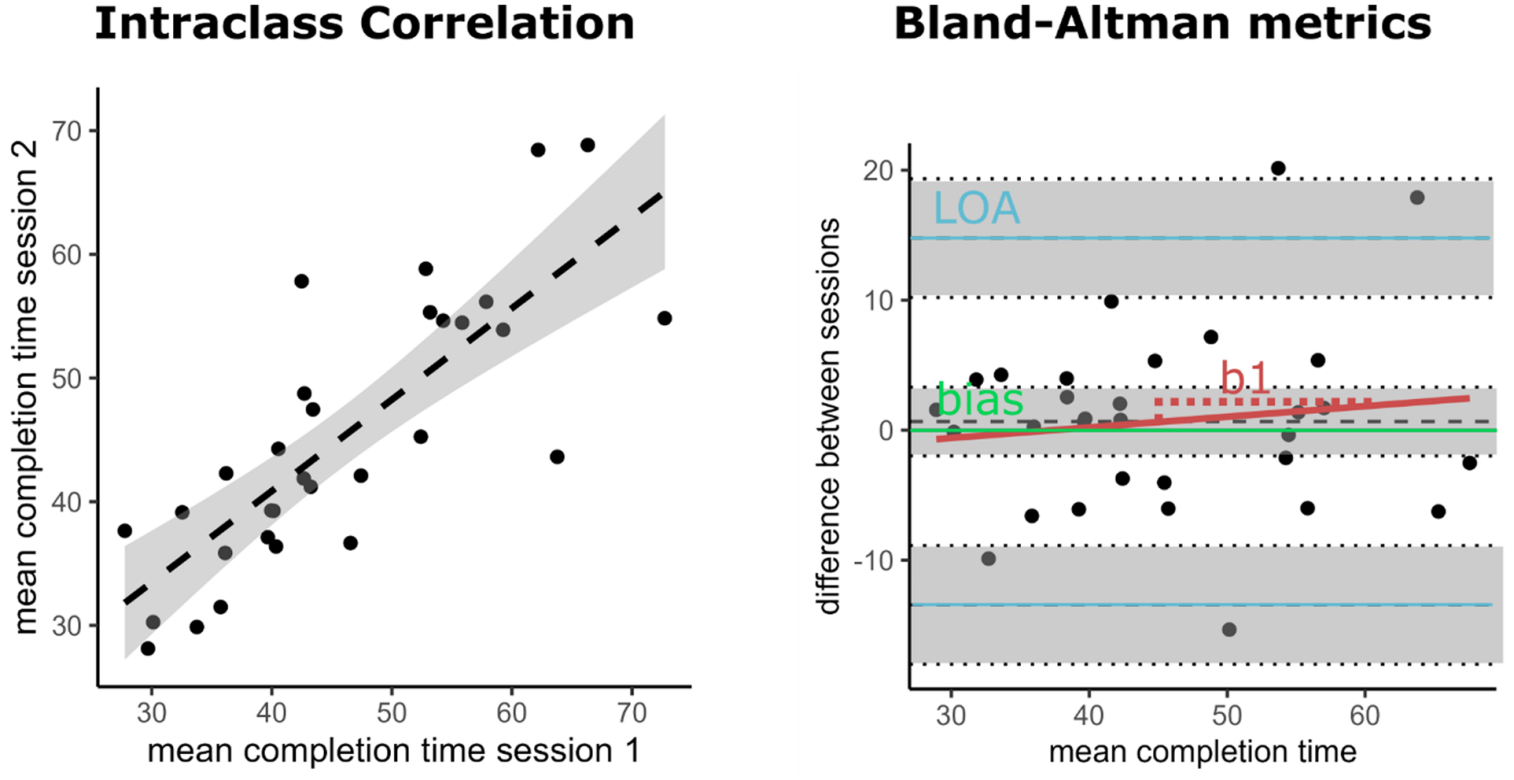
Illustrative example of chosen reliability metrics. The left panel shows an approximate depiction (i.e., dashed line) of an intraclass correlation representing the relative agreement between two sessions. The right panel shows a Bland–Altman plot including its descriptive metrics representing the absolute agreement between sessions. The bias is the mean difference between sessions across all participants. The limits of agreement (LOAs) mark the values that contain 95% of the individual differences between sessions. The weight of the regression (*b*_1_ highlighted in red) from difference between sessions ∼ mean completion time can uncover systematic differences arising between sessions. Gray areas represent 95% CIs. The illustrated data represent the accuracy condition in TMT-A of sample A.

All trials were included in the analysis. We analyzed reliabilities across samples in two ways (see also Figure 2 for a visualization of the different metrics). First, we calculated the ICCs and determined 95% confidence intervals (CIs) based on a two-way, mixed-effects model for average agreement (ICC[A,2]) (cf. Koo & Li, 2016). We used this model because we were interested in the test–retest reliability for a task where participants performed more than one trial in each investigated condition. ICCs were calculated for each experimental condition ([TMT-A] TMT-B; speed and accuracy instructions) in sample A and sample B. Classifications of ICCs are as follows: excellent (>0.80), good (0.60–0.80), moderate (0.40–0.60), or poor (<0.40) (Cicchetti & Sparrow, 1981; Clark et al., 2022; Landis & Koch, 1977). The results provide an estimate of the relative stability of the score in terms of its interindividual stability. Second, we provide measures based on Bland–Altman analyses of the absolute agreement of two measures (Bland & Altman, 1999). The bias describes the mean difference between the two measures. Because the value is computed by subtracting measure two from measure one, in our case positive values indicate larger measures in session one of a sample. Thus, the bias describes the absolute stability of a test score. A bias of zero would indicate that on average a test score did not change across sessions. The limits of agreement (LOAs) specify the range covering 95% of the measured differences between one and two. That is, although the absolute stability of a score could be good (e.g., its bias is zero), individual measures could still disperse widely around this bias. The LOAs therefore indicate how stable the bias is across the sample. And, finally, we provide the fixed slope of the Bland–Altman analysis—that is, the slope of the linear regression predicting the difference between measures by the mean of measures. This gives an estimate of possible systematic differences between time points which might be characteristic of specific ranges of variables (e.g., participants that are slow on average might differ less between time points). To facilitate qualitative conclusions about these systematic differences, we report the significance of the slope. That is, if a slope is significant, there is a systematic relationship between individuals’ performances and across-session effects. Analyses were conducted in R 4.1.3 (R Core Team, 2019). For computation of the ICCs, we used package irr (Gamer, Lemon, & Singh, 2019). For the computation of Bland–Altman statistics, we used the package blandr (Datta, 2017). In terms of slopes of the Bland–Altman regression we report results as significant if *p* < 0.05; there were no adjustments for multiple comparisons.

## Results and discussion

The results for sample A, including the respective measures of reliability and an overview of the descriptive statistics for each test score for the initial test, as well as the retest, are given in Table 1. Table 2 contains the same results for sample B.

**Table 1.** Sample A results on test–retest reliabilities of scores in the computerized TMT. Sessions were 3 days apart. Results for the Bland–Altman analyses including the bias, the limits of agreement (LOAs; i.e., bias ± 1.96 × standard deviation) and the slope (*b*_1_), and the ICCs based on a two-way, mixed-effects model for average agreement (ICC[A,2]) are given for each investigated test score. For biases and ICCs, 95% CIs are given in squared brackets. For LOAs, the standard errors of the LOAs are given in parentheses. Slopes printed in bold indicate significant weights (*p < 0.05, **p < 0.01, ***p < 0.001).

			Descriptive statistics		Bland–Altman			
DV	Condition		Session 1	Session 2 (3 d later)	Bias	LOAs	*b*_1_	ICC (A,2)
Completion time (s)	A	Speed	36.89 (9.04)	35.16 (7.39)	–0.32 [–2.08 to 1.44]	–9.71 to 9.08 (1.49)	0.10	0.82 [0.70–0.89]
		Accuracy	52.04 (18.63)	53.66 (18.79)	0.66 [–1.98 to 3.30]	–13.44 to 14.77 (2.23)	0.08	0.86 [0.77–0.92]
	B	Speed	52.06 (12.94)	47.34 (11.60)	4.12 [1.15–7.10]	–11.77 to 20.01 (2.52)	0.33*	0.70 [0.47–0.82]
		Accuracy	66.43 (22.27)	59.21 (18.09)	2.49 [–0.57 to 5.54]	–13.54 to 18.83 (2.59)	–0.02	0.64 [0.41–0.78]
Speed–accuracy measure	A	Speed	0.03 (0.26)	0.05 (0.31)	–0.01 [–0.13 to 0.10]	–0.64 to 0.62 (0.10)	–0.24	0.06 [0.00–0.43]
(log odd accuracy ∼ rt)		Accuracy	0.04 (0.22)	0.04 (0.21)	0.00 [–0.06 to 0.06]	–0.34 to 0.34 (0.05)	0.04	0.26 [0.00–0.55]
	B	Speed	0.02 (0.18)	0.12 (0.20)	–0.10 [–0.18 to 0.03]	–0.53 to 0.45 (0.07)	0.10	0.00 [0.00–0.26]
		Accuracy	0.01 (0.15)	0.03 (0.14)	–0.03 [–0.07 to 0.02]	–0.28 to 0.23 (0.04)	–0.13	0.26 [0.00–0.55]
Fixation duration (ms)	A	Speed	174.26 (15.01)	177.20 (16.86)	0.69 [–3.81 to 5.20]	–22.93 to 24.31 (3.80)	–0.08	0.84 [0.73–0.90]
		Accuracy	172.51 (17.60)	177.78 (15.42)	–2.06 [–6.51 to 2.39]	–25.40 to 21.29 (3.76)	–0.13	0.85 [0.76–0.91]
	B	Speed	178.32 (17.99)	179.09 (16.43)	–0.12 [–3.99 to 3.75]	–20.42 to 20.19 (3.27)	–0.03	0.91 [0.85–0.95]
		Accuracy	176.96 (18.25)	180.65 (17.20)	1.55 [–3.12 to 6.22]	–22.97 to 26.07 (3.95)	0.05	0.84 [0.74–0.91]
Saccade amplitude (°)	A	Speed	4.37 (0.66)	4.36 (0.55)	0.08 [–0.11 to 0.27]	–0.92 to 1.07 (0.16)	–0.18	0.76 [0.60–0.86]
		Accuracy	4.12 (0.71)	4.03 (0.60)	–0.11 [–0.29 to 0.08]	–1.08 to 0.87 (0.16)	–0.44**	0.74 [0.57–0.84]
	B	Speed	4.34 (0.62)	4.28 (0.64)	–0.00 [–0.14 to 0–14]	–0.71 to 0.71 (0.11)	–0.07	0.79 [0.64–0.87]
		Accuracy	4.22 (0.66)	4.19 (0.58)	–0.02 [–0.18 to 0.14]	–0.85 to 0.82 (0.13)	0.03	0.82 [0.69–0.89]
*N* fixations								
All	A	Speed	138.03 (35.12)	129.79 (26.65)	4.95 [–3.39 to 13.29]	–38.80 to 48.70 (7.04)	00.20	0.73 [0.55–0.84]
		Accuracy	150.41 (42.34)	144.01 (36.45)	8.10 [–0.87 to 17.07]	–39.98 to 55.18 (7.58)	–00.04	0.69 [0.48–0.82]
	B	Speed	190.39 (45.98)	172.24 (40.25)	15.22 [4.04 to 26.39]	–43.43 to 73.86 (9.44)	00.37*	0.77 [0.53–0.88]
		Accuracy	198.76 (56.20)	180.53 (50.26)	7.63 [0.05 to 15.22]	–32.18 to 47.45 (6.41)	–00.15	0.61 [0.34–0.76]
Guiding	A	Speed	14.92 (3.73)	13.76 (3.42)	0.02 [–1.42 to 1.46]	–6.97 to 7.01 (1.21)	–00.03	0.22 [0.00–0.55]
		Accuracy	16.00 (3.86)	14.41 (3.74)	0.46 [–1.00 to 1.92]	–6.63 to 7.56 (1.23)	–00.15	0.41 [0.00–0.66]
	B	Speed	19.14 (4.31)	17.30 (4.22)	0.77 [–0.87 to 2.41]	–7.18 to 8.72 (1.38)	00.42	0.30 [0.00–0.60]
		Accuracy	18.71 (5.21)	17.92 (4.58)	0.31 [–0.90 to 1.51]	–5.55 to 6.16 (1.01)	00.01	0.32 [0.00–0.61]
Searching	A	Speed	121.42 (27.79)	115.02 (21.40)	5.29 [–3.60 to 14.17]	–37.82 to 48.40 (7.47)	00.29	0.63 [0.37–0.79]
		Accuracy	133.09 (34.31)	127.61 (31.11)	8.25 [–1.29 to 17–79]	–38.05 to 54.55 (8.02)	–00.12	0.56 [0.24–0.74]
	B	Speed	169.30 (33.41)	151.00 (33.45)	16.88 [4.80 to 28.97]	–41.78 to 75.55 (10.16)	00.77***	0.48 [0.07–0.70]
		Accuracy	177.77 (45.95)	158.48 (41.39)	8.31 [1.07 to 15.55]	–26.83 to 43.44 (6.09)	–00.16	0.66 [0.41–0.80]
Eye–hand span (ms)	A	Speed	977.11 (302.58)	1007.48 (308.68)	–9.34 [–80.62 to 61.94]	–355.22 to 336.54 (59.92)	0.02	0.31 [0.00–0.61]
		Accuracy	1529.05 (723.59)	1654.65 (652.22)	–25.23 [–179.44 to 128.99]	–773.56 to 723.11 (129.64)	0.37*	0.74 [0.54–0.85]
	B	Speed	1269.94 (339.61)	1167.97 (388.91)	110.56 [–17.00 to 238.12]	–508.44 to 729.56 (107.23)	0.70**	0.49 [0.12–0.70]
		Accuracy	1782.96 (714.45)	1643.85 (545.68)	12.35 [–115.80 to 140.49]	–609.94 to 856.05 (107.73)	0.06	0.69 [0.45–0.82]
Scanpath (°)	A	Speed	729.08 (194.35)	693.24 (146.57)	31.48 [–22.09 to 85.06]	–249.73 to 312.70 (45.27)	0.33	0.62 [0.37–0.77]
		Accuracy	768.85 (221.26)	719.31 (170.48)	30.36 [–13.73 to 74.45]	–201.06 to 261.78 (37.26)	–0.37	0.65 [0.41–0.79]
	B	Speed	1005.92 (261.28)	904.22 (226.32)	72.04 [3.52–140.56]	–287.62 to 431.71 (57.90)	0.40*	0.65 [0.41–0.79]
		Accuracy	1014.83 (279.19)	910.78 (254.67)	45.39 [3.50–87.28]	–174.50 to 265.28 (35.40)	–0.06	0.54 [0.24–0.73]

**Table 2.** Sample B results for test–retest reliabilities of scores in the computerized TMT. Sessions were 10 to 30 days apart. Results for the Bland–Altman analyses including the bias, the limits of agreement (LOAs; i.e., bias ± 1.96 × standard deviation) and the slope (*b*_1_), and the ICCs based on a two-way, mixed-effects model for average agreement (ICC[A,2]) are given for each investigated test score. For biases and ICCs, 95% CIs are given in squared brackets. For LOAs, the standard errors of the LOAs are given in parentheses. Slopes printed in bold indicate significant weights (*p < 0.05, **p < 0.01, ***p < 0.001).

			Descriptive statistics		Bland–Altman			
DV	Condition		Session 1	Session 2 (10–30 d later)	Bias	LOAs	*b*_1_	ICC(A,2)
Completion time (s)	A	Speed	33.93 (7.90)	34.25 (7.46)	1.61 [–0.26 to 3.49]	–8.77 to 12.00 (1.59)	0.17	0.71 [0.53–0.82]
		Accuracy	45.86 (12.05)	45.20 (11.12)	–1.62 [–4.38 to 1.14]	–16.85 to 13.62 (2.34)	–0.08	0.91 [0.85–0.94]
	B	Speed	48.39 (13.17)	44.27 (9.88)	4.71 [2.02–7.41]	–10.17 to 19.61 (2.28)	0.02	0.71 [0.48–0.83]
		Accuracy	56.01 (12.60)	53.53 (12.70)	7.23 [3.06–11.39]	–15.78 to 30.25 (3.53)	0.27*	0.82 [0.64–0.90]
Speed–accuracy measure	A	Speed	–0.00 (0.23)	0.15 (0.34)	–0.16 [–0.28 to 0.04]	–0.80 to 0.49 (0.10)	–1.1**	0.12 [0.00–0.44]
(log odd accuracy ∼ rt)		Accuracy	0.05 (0.27)	0.09 (0.26)	–0.04 [–0.13 to 0.04]	–0.50 to 0.41 (0.07)	–0.09	0.48 [0.14–0.69]
	B	Speed	0.05 (0.24)	0.06 (0.18)	–0.02 [–0.10 to 0.07]	–0.47 to 0.43 (0.07)	0.75	0.00 [0.00–0.05]
		Accuracy	0.05 (0.16)	0.06 (0.16)	–0.01 [–0.07 to 0.05]	–0.34 to 0.32 (0.05)	–0.82	0.17 [0.00–0.50]
Fixation duration (ms)	A	Speed	182.61 (20.80)	181.43 (20.50)	–4.16 [–6.95 to 1.37]	–19.32 to 11.01 (2.36)	–0.04	0.82 [0.69–0.89]
		Accuracy	182.40 (17.17)	185.30 (22.02)	–4.89 [–8.35 to 1.43]	–24.02 to 14.24 (2.93)	0.13	0.83 [0.70–0.90]
	B	Speed	183.70 (21.37)	184.00 (21.64)	–1.08 [–3.85 to 1.70]	–16.42 to 14.27 (2.35)	0.09	0.91 [0.85–0.94]
		Accuracy	184.78 (21.72)	183.94 (21.35)	–2.91 [–6.13 to 0.32]	–20.74 to 14.92 (2.73)	0.12	0.90 [0.83–0.94]
Saccade amplitude (°)	A	Speed	4.57 (0.68)	4.49 (0.77)	0.03 [–0.13 to 0.19]	–0.84 to 0.90 (0.08)	0.27	0.75 [0.60–0.85]
		Accuracy	4.27 (0.59)	4.37 (0.84)	0.09 [–0.09 to 0.26]	–0.87 to 1.05 (0.15)	0.22	0.65 [0.43–0.79]
	B	Speed	4.48 (0.64)	4.48 (0.64)	0.05 [–0.07 to 0.18]	–0.65 to 0.75 (0.11)	–0.01	0.82 [0.71–0.89]
		Accuracy	4.31 (0.67)	4.33 (0.65)	0.03 [–0.08 to 0.15]	–0.60 to 0.67 (0.10)	0.15	0.83 [0.72–0.90]
*N* fixations								
All	A	Speed	126.18 (31.71)	122.18 (27.39)	8.72 [1.12–16.32]	–32.59 to 50.03 (6.43)	0.31*	0.68 [0.47–0.81]
		Accuracy	133.09 (28.65)	125.76 (29.96)	6.64 [–1.73 to 15.00]	–39.61 to 52.88 (7.09)	0.10	0.80 [0.68–0.88]
	B	Speed	167.80 (45.86)	153.14 (33.03)	19.59 [10.17–29.01]	–32.49 to 71.67 (7.99)	0.01	0.68 [0.40–0.82]
		Accuracy	168.73 (39.63)	160.11 (38.96)	20.24 [6.89–33.59]	–53.22 to 94.04 (11.32)	0.14	0.71 [0.48–0.83]
Guiding	A	Speed	12.51 (3.04)	12.83 (3.00)	1.33 [–0.15 to 2.81]	–6.72 to 9.37 (1.25)	0.16	0.43 [0.08–0.65]
		Accuracy	13.93 (3.28)	13.75 (3.45)	1.59 [0.25–2.93]	–5.81 to 9.00 (1.14)	0.04	0.58 [0.32–0.75]
	B	Speed	16.45 (4.29)	15.81 (3.05)	1.83 [0.33–3.34]	–6.47 to 10.14 (1.27)	0.03	0.36 [0.00–0.60]
		Accuracy	16.80 (3.74)	16.77 (3.29)	0.79 [–0.69 to 2.27]	–7.39 to 8.96 (1.25)	0.16	0.61 [0.37–0.76]
Searching	A	Speed	109.93 (27.26)	106.37 (20.62)	6.59 [–0.49 to 13.68]	–31.92 to 45.11 (6.00)	0.29	0.66 [0.44–0.79]
		Accuracy	115.63 (24.26)	109.49 (24.41)	5.48 [–1.97 to 12.94]	–35.73 to 46.70 (6.32)	0.11	0.80 [0.68–0.88]
	B	Speed	148.35 (36.56)	133.85 (18.74)	18.30 [9.60–27.00]	–29.79 to 66.39 (7.37)	–0.00	0.68 [0.38–0.82]
		Accuracy	148.00 (28.56)	140.73 (29.12)	19.29 [6.73–31.85]	–50.15 to 88.72 (10.65)	0.12	0.67 [0.43–0.81]
Eye–hand span (ms)	A	Speed	958.48 (264.24)	967.82 (267.08)	–39.00 [–123.59 to 45.60]	–498.87 to 420.88 (71.63)	0.06	0.66 [0.44–0.79]
		Accuracy	1362.76 (564.40)	1387.98 (458.02)	–125.59 [–260.79 to 9.60]	–872.92 to 621.73 (114.59)	0.05	0.81 [0.68–0.88]
	B	Speed	1210.79 (402.98)	1100.23 (271.48)	101.96 [20.15–183.78]	–350.27 to 554.19 (69.34)	–0.08	0.42 [0.06–0.64]
		Accuracy	1515.09 (423.44)	1502.74 (415.14)	139.11 [–29.04 to 307.26]	–790.34 to 1068.56 (142.52)	0.39*	0.70 [0.52–0.82]
Scanpath (°)	A	Speed	696.0502 (188.66)	664.57 (150.82)	40.51 [–6.43 to 87.45]	–214.68 to 295.69 (39.75)	0.41*	0.57 [0.30–0.74]
		Accuracy	692.6145 (134.72)	662.25 (168.42)	49.54 [–7.06 to 106.15]	–263.38 to 362.46 (47.98)	0.39*	0.56 [0.28–0.73]
	B	Speed	922.24 (271.67)	850.20 (199.84)	101.70 [41.92–161.48]	–228.73 to 432.14 (50.67)	0.10	0.61 [0.34–0.77]
		Accuracy	886.96 (221.13)	841.578 (216.46)	104.04 [26.00–182.08]	–327.33 to 535.41 (66.15)	0.08	0.61 [0.34–0.77]

### Precision estimation

Given that we aimed to describe reliabilities through estimates of a coefficient (i.e., ICCs) rather than test coefficients against some value, we examined the precision of our estimation. Therefore, we calculated the widths of the 95% CIs that we could achieve given our sample sizes for a selection of ICCs (Clark et al., 2022; Doros & Lew, 2010). Adapting the procedure of Clark et al. (2022), we simulated the data of 100,000 individuals with a predefined ICC. We then estimated the resulting CIs 10,000 times for our sample sizes (i.e., 30 in sample A and 34 in sample B). The results depicted in Table 3 demonstrate that the CIs were wide for low ICCs but narrower for higher ICCs. Because we predominantly wanted to identify test scores with high test–retest reliabilities, our samples should provide a valid assessment for this aim. Furthermore, the examination of two independent samples with differing test–retest intervals is beneficial because the time between testing occasions can be a critical factor (Mollon et al., 2017). Similar results in both samples therefore improve the estimation of test-retest reliabiltiy.

**Table 3.** Precision estimations for given sample sizes. Based on the range of 95% CIs for three assumed values of ICCs we should be able to find, given the sample sizes of sample A (*n* = 30) and sample B (*n* = 33).

True ICC	Sample size, *n*	95% CI
0.4	31	0.05–0.78
	34	0.08–0.77
0.6	31	0.44–0.87
	34	0.46–0.87
0.8	31	0.75–0.94
	34	0.76–0.94

### Completion time

As expected, completion times were generally faster in TMT-A than in TMT-B and slower in conditions emphasizing accuracy compared with speed. As evident by the biases, performance in TMT-A was relatively stable across sessions, whereas completion times in TMT-B tended to be faster in later sessions, irrespective of times between sessions and spread across a wider range (cf. Tables 1 and 2). This parallels results on training effects studied in the TMT indicating that performance improves with repeated testing, especially in TMT-B (Buck, Atkinson, & Ryan, 2008; McCaffrey, Ortega, & Haase, 1993). Also, we found a significantly positive slope in TMT-B for speed-instruction completion times in Sample B and accuracy-instruction completion times in Sample A respectively, suggesting that slower participants showed larger training effects. However, these results were only present in one sample respectively and not consistent across time points. Results for the ICCs indicated moderate to excellent test–retest reliabilities with TMT-A (0.71 ≤ ICC ≤ 0.91), which provided more stable results than TMT-B (0.64 ≤ ICC ≤ 0.82). This result is comparable with ranges found in other versions of the TMT (Bracken, Mazur-Mosiewicz, & Glazek, 2019; Park & Schott, 2021), although slightly worse than expected for TMT-B.

### Speed–accuracy measure

The new speed–accuracy score as a measure of internal cognitive control yielded interesting results. Surprisingly, the measure was relatively constant across conditions. Participants did not differ in their speed–accuracy trade-offs whether they emphasized speed or the accuracy of their actions. Additional analyses indicated that they indeed performed more accurately or quickly in the respective conditions. However, their overall trade-off represented in our chosen measure exhibited no difference. Unfortunately, this seemingly locked setting did not represent trait-like characteristics; test–retest reliabilities in both samples were poor (0.00 ≤ ICC ≤ 0.48).

### Fixation duration

Fixation durations were stable across all conditions and time points. The absolute agreement across sessions was constant, and we found no indication of relevant practice effects or systematic biases. Also, we found ICCs that could be classified as excellent in every condition (0.82 ≤ ICC ≤ 0.91).

### Saccade amplitude

As with the fixation durations, saccade amplitudes gave stable results across all time points. Except for the speed condition in TMT-A at T2, there was no indication of practice effects or systematic biases. Here, a negative slope suggests that participants with smaller amplitudes across sessions performed larger eye movements on their second session (*b*_1_ = –0.44). However, because this effect was only present in one condition at one time point, we cannot infer any regularities. Overall, saccadic amplitudes showed good test–retest reliability in TMT-A (0.65 ≤ ICC ≤ 0.76) and good to excellent test–retest reliability in TMT-B (0.79 ≤ ICC ≤ 0.83).

### Number of fixations

Participants performed more fixations in TMT-B than in TMT-A. This finding is consistent with previous studies on eye tracking in the TMT (Hicks et al., 2013; Linari et al., 2022; Recker et al., 2022). Across all conditions, we furthermore found a practice effect in later sessions, especially pronounced in TMT-B. Two significant slopes of the Bland–Altman regression in the speeded conditions (sample B–TMT-A; sample A–TMT-B) indicated that this practice effect was larger for participants with overall higher numbers of fixations. Still, the ICCs indicate good test–retest reliabilities across conditions (0.61 ≤ ICC ≤ 0.80).

### Number of guiding fixations

We found a slightly greater number of guiding fixations (i.e., fixations on the current target of the sequence) in TMT-B than in TMT-A. Across sessions, the number remained relatively constant; however, the ICC results indicate no interindividual stability of the number across multiple sessions (0.22 ≤ ICC ≤ 0.61). Comparing this finding with earlier studies, the absolute stability signaled by the Bland–Altman statistics resembles the stabilities across testing conditions found by Recker et al. (2022). Considering the overall low number of guiding fixations could hinder this absolute stability translating to the interindividual level. Because the range of the measure is restricted, the results cannot reliably differentiate between persons (Dietze, Recker, & Poth, 2023; Hedge et al., 2018).

### Number of searching fixations

The number of searching fixations provided results similar to those for the overall number of fixations. Participants performed more searching fixations(i.e., time locating future targets) in TMT-B than in TMT-A. Repeated sessions also led to less of these fixations, especially in TMT-B. Furthermore, we found a significant slope indicating larger learning effects for participants with overall greater numbers of searching fixations in the speeded condition of TMT-B in sample A. ICCs were also comparable with the values found for the overall number of fixations, but they were slightly worse and more dispersed. Values ranged from moderate to good (0.48 ≤ ICC ≤ 0.80).

### Eye–hand span

The eye–hand span, indicating the temporal distance between eye and hand/cursor movements, was comparable between TMT-A and -B but expectedly longer when participants were instructed to emphasize accuracy over speed. Results on the stability across sessions, however, were quite diverse. Seemingly, eye–hand spans got longer in repeated sessions of TMT-A but they decreased in TMT-B. However, in both test halves, the range of values was comparably large. This mixed picture of results was confirmed by the results on test–retest reliabilities in terms of the ICCs. Eye–hand spans in the accuracy trials were overall more reliable, with good to excellent ICCs (0.69 ≤ ICC ≤ 0.81). In the speeded trials, these values ranged from poor to moderate (0.31 ≤ ICC ≤ 0.66). Finally, regression within the Bland–Altman statistics indicated three instances of possibly systematic biases in practice effects. That is, participants with overall larger eye–hand spans tended to show smaller spans in repeated sessions in certain conditions.

### Scanpath length

The overall path participants covered with their eye movements was again greater in TMT-B than in TMT-A. Also, the greater practice effects in the TMT-B that were evident in the earlier test scores were repeated here. The ICCs were comparable across all conditions, with moderate to good values ranging between 0.54 and 0.65. Here, also, there was a tendency for systematic biases in three conditions, indicating larger practice effects for participants with overall longer scanpaths.

## General discussion

**Figure 3.**
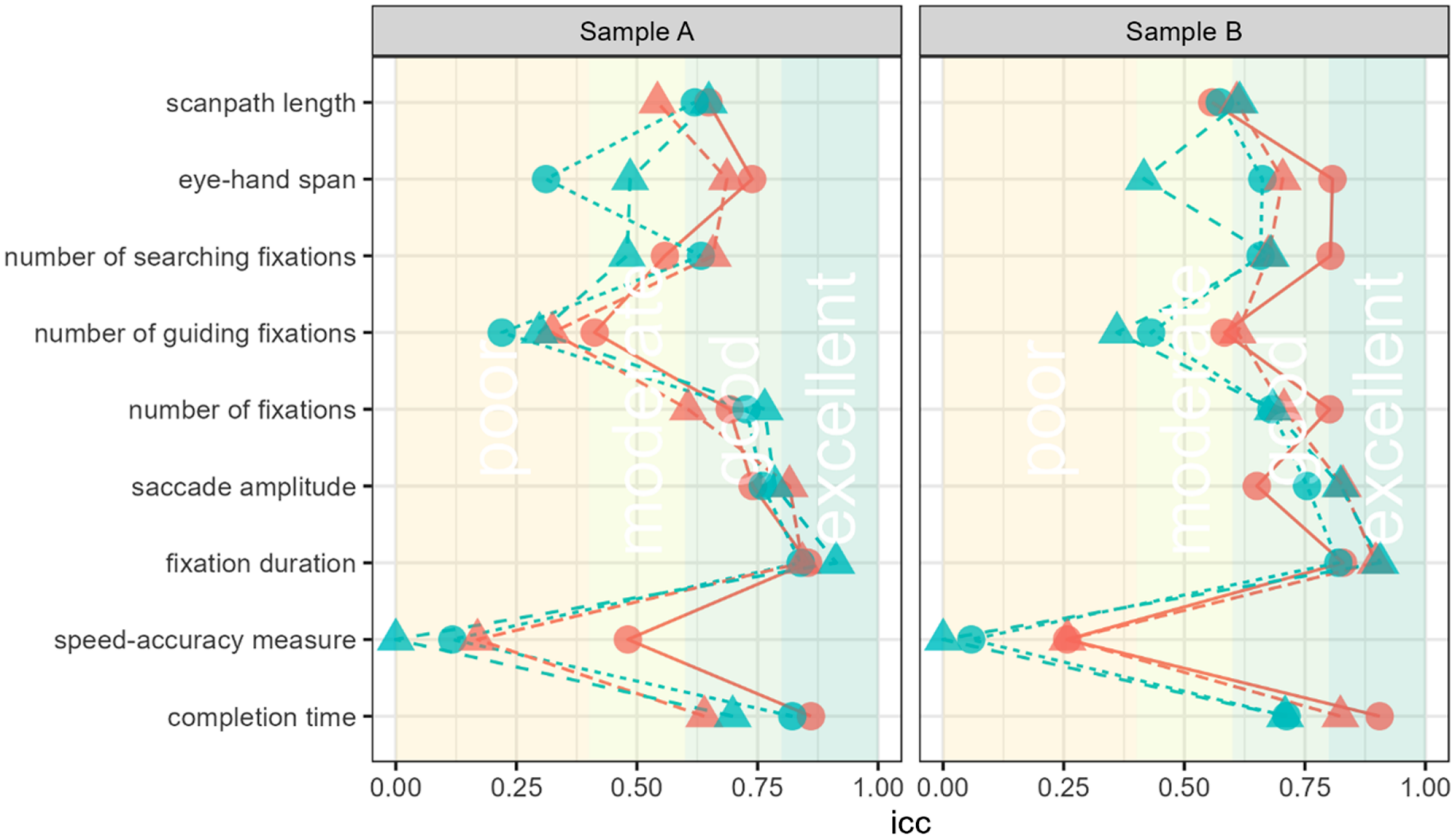
Intraclass correlations for dependent variables examined in the TMT. Results for the intraclass correlations for each examined score in the TMT after 3 days (sample A) and 10 to 30 days (sample B). Circles indicate results for TMT-A, triangles indicate results for TMT-B, blue indicates speed instructions, red indicates accuracy instructions, and the vertically written and color-delimited classifications are according to Landis & Koch (1977).

The aim of the present study was to assess the test–retest reliabilities of different test scores in an eye-tracking version of the TMT. Cognitive assessments using the most frequently applied version of the TMT mostly rely on the examination of completion times in the two test halves A and B (Bowie & Harvey, 2006). However, considering the number of affected cognitive functions that contribute to test performance the specificity of conclusions based on the usually provided test scores is limited. Recent studies demonstrated how scores based on eye movements can shed light on specifics that determine differences in observed performances (Linari et al., 2022; Recker et al., 2022; Wölwer & Gaebel, 2002). Using a recently introduced version of the TMT by Recker et al. (2022), we examined whether scores of this test version not only increase our general understanding of the TMT but also capture reliable interindividual differences with regard to their stability over time (for a summary of the estimated ICCs, see Figure 3). First, we found that the reliabilities of completion times were good to excellent, comparable with earlier versions of the test. Of the additionally included eye-tracking test scores, two provided good to excellent reliabilities (i.e., fixation durations and saccade amplitudes), three provided moderate to good reliabilities (i.e., number of fixations, number of searching fixations, and scanpath length), and the rest presented mixed results depending on experimental conditions (i.e., number of guiding fixations and eye–hand spans).

Based on speed–accuracy instructions included in the test, we computed a test score to provide a measure of internal cognitive control. In contrast to the often-used difference score between test halves, this measure should present a stimulus-independent estimate of the participants’ cognitive control based on their ability to shift between task sets. However, results for this speed–accuracy measure were not consistent in the sense that the measure was stable over time but neither differentiated between conditions nor produced reliable individual differences. This suggests that if the speed–accuracy trade-off was a personal trait, then the absence of interindividual stability should have been due to range restrictions. Thus, to conclude, the speed–accuracy trade-off score offers an interesting new measure for longitudinal assessments. However, it remains a question for future studies as to whether it can be used for individual diagnostics, as well.

Of the investigated eye movement scores, fixation durations and saccade amplitudes presented the most reliable results in terms of stability of scores across time points and the found interindividual differences. Both measures are frequently examined in various contexts ranging from reading (Rayner, 1998) to the exploration of natural scenes (Dorr, Martinetz, Gegenfurtner, & Barth, 2010) to the performance of everyday tasks (Land & Tatler, 2009); that is, they can be associated with a multitude of different functions related to information accumulation processes. For example, fixation durations can be reflective of ongoing information extraction and action planning, and the length of saccades often varies depending on the context given by task sets (e.g., Hutton, 2008; Liversedge & Findlay, 2000; Mills, Hollingworth, Van der Stigchel, Hoffman, & Dodd, 2011; Salthouse & Ellis, 1980). However, both fixation duration and saccade amplitude have been shown to provide interindividually stable results across multiple tasks (Andrews & Coppola, 1999; Poynter, Barber, Inman, & Wiggins, 2013; Rayner et al., 2007); that is, they provide a seemingly stable measure of interindividual differences even across long periods of time (Henderson & Luke, 2014). Our results indicate that this stability is also present in sequential actions, a type of task previously not included in the examination of stabilities for these measures. However, in case of this investigated task, this stability also means that both measures did not vary across conditions; that is, differences between test halves or instructions did not manifest in participants’ fixation durations or saccadic amplitudes. In studies assessing fixation durations and saccadic amplitudes in the TMT, so far only healthy samples have been investigated (Linari et al., 2022; Recker et al., 2022). Studies on clinical populations could provide cases where the interindividual stability of these measures proves useful, as differences in eye movement profiles have been connected to working memory capacities (Luke, Darowski, & Gale, 2018) or intelligence (Hayes & Henderson, 2018).

Previous studies investigating differences in eye movements during performance of the TMT have repeatedly found that the number of fixations varies between TMT-A and TMT-B. Furthermore, these studies have pointed out that these differences were due to prolonged periods of orienting, such as increased number of searching fixations (Recker et al., 2022) or planning periods (Linari et al., 2022; Wölwer & Gaebel, 2002). Our results on the test–retest reliabilities of related measures (i.e., number of fixations, number of searching fixations, and the scanpath length) indicate that these differences to some degree can also reliably differentiate among persons. However, we also found that, with repeated testing, they were subject to training effects. Considering their relationship with the overall performance in terms of completion times, this parallels findings that completion times of the TMT, especially TMT-B, improve with repeated testing (Buck et al., 2008; McCaffrey et al., 1993). For one thing, participants’ performance improved because they were more acquainted with the task in their second session. Also, because spatial configurations of the stimuli were repeated between session 1 and session 2, the participants’ memory of the arrangements might also have contributed to these training effects. Beyond completion times, such practice effects could also lead to a homogenization of eye movement behavior (Foerster, 2018; Foerster et al., 2011), which in turn leads to less reliable interindividual differences.

The eye–hand span presented variable results on test–retest reliability depending on the given instruction to emphasize speed or accuracy. The score varied with the speed–accuracy instructions and presented more interindividually stable results in the accuracy condition than in the speed condition. That is, it produced stable results that differentiated between conditions but mixed results with regard to differentiating between persons. This seeming contradiction between the ability to differentiate between experimental conditions but not persons has been described as the “reliability paradox” (Hedge et al., 2018). Eye movement and related cognitive research offer paradigms that robustly produce experimental effects. For example, paradigms such as the Stroop task (e.g., MacLeod, 1991) produce stable effects for studying response interferences in multiple domains. Because of this robustness, the mechanisms behind such effects are well understood and often supported by accompanying cognitive and neurophysiological theories. Therefore, using these theoretical foundations for differential examinations on an individual level could yield great benefits. However, the experimental paradigms used to study these effects often produce no reliable interindividual differences because they are designed to minimize these differences to produce stable effects for the average observer. That is, without a certain degree of interindividual variability, a task might produce stable experimental effects but cannot reliably differentiate between persons. Our results on the eye–hand span illustrate the importance of examining the test–retest reliabilities of scores when considering their possible use for the study of interindividual differences. Not only can one measure differentiate well between conditions but not between persons, but the former can also vary between examined conditions. In this way, considering the test–retest reliability one might also discover which condition to use when asking questions about either underlying cognitive processes or interindividual differences (Clark et al., 2022).

In their study on eye movements in the TMT, Recker et al. (2022) used the additionally introduced manipulation of speed–accuracy instructions to test the degree of interindividual variability of the new eye-tracking scores. They benchmarked the individual variability in each score against the variability introduced by the strong, ubiquitous manipulation that is the speed–accuracy trade-off. If variability in a score was dominated by the manipulation, then it was dubbed “experimentally dominated.” In contrast, if an interindividual difference exceeded the variability introduced by the manipulation, then it was dubbed “individually dominated.” Similar to the examination of test–retest reliabilities, this classification helps to identify which scores might be best suited to answer which kinds of questions. However, because it always depicts scores relative to the condition chosen as a benchmark it provides a form of convergent or divergent validity rather than reliability. In this way, our results on the test–retest reliabilities of the examined scores corroborate the analysis of the original study. For example, we showed that not only are fixation durations and saccade amplitudes individually dominated compared to speed-accuracy emphases as found by Recker et al. (2022), but, on top of that, they are also a stable source of interindividual differences across multiple sessions. On the other hand, we can also see that scores reflective of speed–accuracy instructions in the original study (e.g., completion times, number of fixations) can still provide good results in terms of their interindividual reliabilities and thus can be used to study differences between persons regarding this construct.

Eye-tracking measures bear ambiguous results for the study of interindividual differences. Although they yield great potential for clinical diagnostics (Itti, 2015) and the use of correlational approaches in experimental psychology (Mollon et al., 2017; Wilmer, 2008) a lot of established tasks from cognitive research and vision science poorly translate to these fields of application (Clark et al., 2022; Hedge et al., 2018). Our results parallel this ambiguity in the sense that they once again demonstrate that certain eye-tracking measures can reliably differentiate among persons and produce stable results even with repeated testing (e.g., fixation duration, saccade amplitude). However, measures that differentiate well between experimental conditions (that is, those that produce stable experimental effects) are less reliable (e.g., number of [searching] fixations) or are possibly too restricted in their range to reliably represent individual differences (e.g., number of guiding fixations and speed–accuracy measure). This highlights the importance of investigating the test–retest reliability of scores when aiming to use them for interindividual difference research and, in our case, ultimately apply them in neuropsychological settings. Accordingly, although not conducted with a clinical sample, the present study is a necessary step toward clinical applications. Results of healthy participants do not necessarily transfer to clinical populations; however, to take paradigms beyond the setting of basic research requires creating the best possible premises for their application. Based on their high data quality, test scores with the most reliable results in healthy samples can be taken as the best candidate test scores to assess in clinical samples. Establishing the reliability of test scores in a healthy sample, therefore, is an important and frequently applied technique to identify the most promising scores to relate to other cognitive and possibly impaired functions and create benchmarks for scores in a healthy sample (Anderson, 2013; Gestefeld, Schneider, & Poth, 2023; Klein & Fischer, 2005; Paap & Sawi, 2016).

## Conclusions

To sum up, the present study has established the test–retest reliability of a TMT that includes eye-tracking scores. Although previous studies indicated the usefulness of examining eye movements within the test to increase the general understanding of the determinants of performance, we provide evidence that some of these scores can also be used to study interindividual differences in these performances. Processes related to information accumulation seem best suited for the study of interindividual differences, such as fixation durations, saccade amplitudes, and number of fixations, whereas processes of eye–hand coordination seem to better differentiate between experimental conditions than between persons (e.g., number of guiding fixations, eye–hand span). In this way, we can increase our understanding of eye movements and possible applications of derived scores in the TMT.

## Footnotes

1 Initially 10 to 14 days were planned as the test-retest interval of sample B. This is also the interval mentioned in the pre-registration of this study. However, because data collection was heavily affected by the covid-19 pandemic, only 21 participants were able to return within 10 – 14 days. Therefore, we decided to extend the interval so that participants could complete their second session at a later point in time. In the end, in sample B 34 participants completed two sessions with a final test-retest interval of 10 – 30 days.
